# Immune monitoring of interleukin-7 compassionate use in a critically ill COVID-19 patient

**DOI:** 10.1038/s41423-020-0516-6

**Published:** 2020-07-29

**Authors:** Guillaume Monneret, Donatien de Marignan, Rémy Coudereau, Céline Bernet, Florence Ader, Emilie Frobert, Morgane Gossez, Sébastien Viel, Fabienne Venet, Florent Wallet

**Affiliations:** 1 Hospices Civils de Lyon, Immunology Laboratory, Edouard Herriot Hospital, Lyon, France; 2 Hospices Civils de Lyon, Lyon-Sud University Hospital, Medical Intensive Care Unit, Lyon, France; 3 Hospices Civils de Lyon, Department of infectious Diseases, North University Hospital, Lyon, France; 4 International Center of Research in Infectiology (CIRI), INSERM U1111, CNRS-UMR 5308, ENS Lyon, Université Claude Bernard Lyon, Lyon University, Lyon, France; 5 Hospices Civils de Lyon, Department of Virology, Infective Agents Institute, North University Hospital, Lyon, France; 6 Hospices Civils de Lyon, Lyon-Sud University Hospital, Immunology Laboratory, 69495 Pierre Bénite, France

**Keywords:** Biomarkers, Diagnostic markers

One of the immune characteristics of coronavirus disease 2019 (COVID-19) is a massive fall in lymphocyte count in which magnitude associates with mortality.^1,2^ Recent monitoring of COVID-19 intensive care units (ICU) patients confirmed the profound lymphopenia and its remarkable stability over time.^3,4^ While most immunomodulation approaches proposed so far in COVID-19 focused on inhibiting inflammatory cytokine response; mounting evidence indicates that this viral-induced defective lymphocyte response may play a central role in COVID-19 pathophysiology.^5^ Interestingly, recombinant human interleukin-7 (IL-7) therapy, known to efficiently restore lymphocyte count in several viral infections was safely administered in septic shock patients^6^ who present with similar lymphocyte alterations as observed in COVID-19.^3^

We report here the case of a 74-year-old patient without any comorbidity. He was admitted to our university hospital ICU (Hospices Civils de Lyon, France) for COVID-19 ARDS requiring high flow oxygen. ICU admission (thereafter corresponding to day 0) occurred 9 days after first symptom onset. SARS-COV-2 PCR was positive (nasal swab) and CT scan was highly suspect of severe COVID-19. He was intubated 24 h after admission and ventilation was set according to guidelines for ARDS including prone positioning. PEEP was around 8–10 cm H_2_O for the whole ICU stay. Antibiotics were initiated at admission until bacterial samples were negative. On day 10, as the patient was still presenting with severe ARDS without any infection criteria, steroids were initiated at 1 mg/kg/day (equivalent prednisolone) but stopped 5 days later (i.e., day 15) due to ventilator associated pneumonia (VAP, *Morganella morganii* and *Aspergillus fumigatus* were identified in BALF). Antibiotics and antifungal therapies were started immediately. At day 20, an additional VAP was suspected (without any bacterial documentation) treated with meropenem. After admission, SARS-CoV-2 PCR remained positive at D10 and D16.

From day 0 to day 24, the patient remained deeply lymphopenic and presented with markedly decreased monocytic expression of HLA-DR (Fig. 1) reflecting deep immunocompromised state. Therefore, at day 24, while the patient did not show any improvement in pulmonary function, presented with several intercurrent infections, absence of negativation of SARS-CoV-2 PCR, and marked and persisting lymphopenia, compassionate use of IL-7 was initiated in order to improve immunity and consequently allow viral clearance. After inaugural injection (3 µg/kg), the patient received IL-7 at 10 µg/kg twice a week during 4 weeks. The patient did not present any serious adverse event except for a transient skin rash at site of injection. Impressive improvement in lymphocyte count and mHLA-DR expression was rapidly observed (Fig. 1). Four days after initiation of IL-7 (day 28), results of SARS-CoV-2 detection by PCR were negative, IFN score started to decrease and circulating IFN-γ returned to normal range. In parallel, clinical condition slowly improved. Mechanical ventilation and sedation were interrupted by day 40 and the patient started to awake and opened his eyes for the first time in 6 weeks. At day 38, the patient developed a Pseudomonas infection successfully treated by ceftazidim and ciprofloxacin. At this time, a transient fall in both lymphocyte count and mHLA-DR was noticed. Both parameters rapidly rose again to normal values after this intercurrent infection. Unfortunately, at day 45, the patient developed an acute kidney injury that was not linked to obstructive or cardiogenic origin. At this stage, in accordance with the patient’s will, his family refused dialysis that was necessary. Treatment was therefore switched to fully palliative care resulting in death of the patient on day 46.

**Fig. 1:**
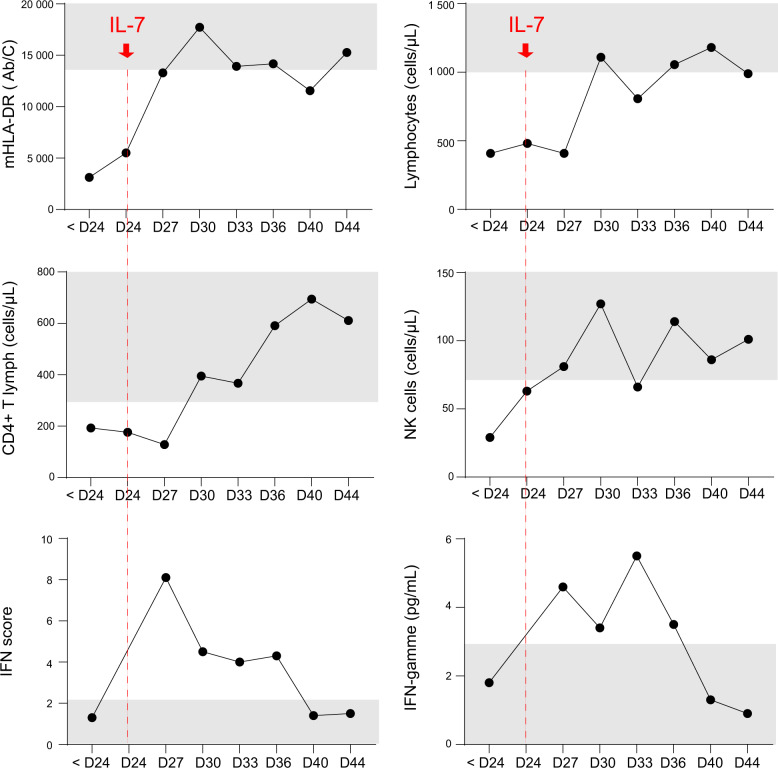
Immunophenotyping overtime. Patient was sampled overtime after ICU admission. Cellular immunophenotyping was performed by flow cytometry and results are expressed as numbers of antibodies bound per monocyte (Ab/C) for monocytic HLA-DR expression and as numbers of cells per µL of whole blood for total lymphocyte, CD4+ T lymphocyte and NK cell counts. Plasma IFNγ concentration was measured by Ella technology and results are expressed as pg/mL. IFN score (type I IFN-related genes mRNA levels) was measured by Nanostring technology and results are expressed as a score relative to normal values. References values (gray zones) are provided by routine clinical immunology laboratories at our institution. Interleukin-7 treatment was initiated at day 24 after ICU admission (D24, red arrow). Cellular measurements were performed once before IL-7 treatment initiation (<D24) and twice a week during 3 weeks after IL-7 treatment initiation: between D27 and D29 (D27), between D30 and D32 (D30), between D33 and D35 (D33), between D36 and D36 (D36), between D40 and D43 (D40), and between D44 and D48 (D44). For each sampling time, if several measurements were performed, the highest value was systematically considered.

Although we cannot draw any definitive conclusion about a single case report, the present results show IL-7 beneficial effects in improving immune functions in a COVID-19 patient. Indeed, after almost 4 weeks in ICU and established protracted immunosuppression, we noticed a marked and rapid elevation of lymphocyte count and mHLA-DR toward reference ranges. This effect was not accompanied by any potential cytokine release of IL-6, IFN-γ, IL-10, or TNF-α (IL-1-β was even never detectable—Supplementary Table S1). Most importantly, this improved immune response was paralleled with negativation of SARS-CoV-2 PCR (and IFN score) and clinical improvement (switching controlled ventilation to pressure support, sedation alleviation, rapid clearance of intercurrent pseudomonas infection).

COVID-19 patients constantly present with severe lymphopenia. Meta-analyses demonstrate the independent association of this immune alteration with poor outcome.^1,2^ In addition, lymphocyte functions were altered in COVID-19.^4^ While viral dissemination is a prominent driver of severe disease, there is mounting evidence suggesting that such altered T-cell function and number may participate in the out of control spiraling of viral replication.^5^ In line, negative correlation between lymphocytes count and pulmonary viral load was observed.^7^ The absence of potent antiviral drug along with severe immune defects therefore contribute to body’s inability to normally eradicate virus^8^ and may explain the long ICU stays reported by many authors. In agreement, first results from autopsy studies reported on the persistence of the virus at the time of death—even after few weeks in ICU.^9^ Another indicator of deep immunosuppression is the extremely high rate of secondary infections in ICU COVID patients. This is especially true for the numerous cases of aspergillosis which are usually seen in very immunocompromised patients. Collectively, there may be a strong rationale for considering drugs aimed at restoring T-cell function and count in the most severe COVID-19 patients. For example, thymosin alpha 1 (which shares similarities with IL-7) recently demonstrated promising results in COVID-19 patients.^10^ The present IL-7 effects, despite unfavorable outcome, appear consistent in terms of immunological recovery as, to date, such a rapid rise in standard immune parameters has not been observed in long-standing COVID-19 patients. In this patient’s case, IL-7 was used in a delayed compassionate manner (after 24 days in the ICU) in a patient likely seriously weakened by a long ICU stay. We may expect better outcome in case of earlier administration. In conclusion, we strongly believe that IL-7 is worth trying in next trials when patients could be stratified based on marked lymphopenia.

## Supplementary information

Table S1
