# Supplementary material for: Immune monitoring of interleukin-7 compassionate use in a critically ill COVID-19 patient
Source: Cell Mol Immunol. 2020 Jul 29;17(9):1001–3. doi: 10.1038/s41423-020-0516-6 (PMC7387803; doi:10.1038/s41423-020-0516-6)
Supplement: Supplementary file 1 — Table S1 [file 41423_2020_516_MOESM1_ESM.docx]

Correspondence – Cellular and Molecular Immunology

**Immune monitoring of interleukin-7 compassionate use in a critically ill COVID-19 patient**

Supplementary material

Guillaume Monneret^1^, Donatien de Marignan^2^, Rémy Coudereau^1^, Céline Bernet^2^, Florence Ader^3,6^, Emilie Frobert^4,6^, Morgane Gossez^1^, Sébastien Viel^5,6^, Fabienne Venet^1^, Florent Wallet^2^

**Affiliations:**

1- Hospices Civils de Lyon, Immunology Laboratory, Edouard Herriot Hospital, Lyon, France

2-Hospices Civils de Lyon, Lyon-Sud University Hospital, Medical Intensive Care Unit, Lyon, France

3- Hospices Civils de Lyon, Department of infectious Diseases, North University Hospital, Lyon, France

4- Hospices Civils de Lyon, Department of Virology, Infective Agents Institute, North University Hospital, Lyon, France

5- Hospices Civils de Lyon, Lyon-Sud University Hospital, Immunology Laboratory, 69495 Pierre Bénite, France

6-International Center of Research in Infectiology (CIRI), INSERM U1111, CNRS-UMR 5308, ENS Lyon, Université Claude Bernard Lyon, Lyon University, Lyon, France

**Corresponding author:**

Prof G. Monneret

Immunology Laboratory, Edouard Herriot Hospital, Hospices Civils de Lyon

Pavillon E - 5 place d’Arsonval - 69437 LYON Cedex 03 - France

E-mail: [guillaume.monneret@chu-lyon.fr](mailto:guillaume.monneret@chu-lyon.fr)

**Table S1. Plasma cytokine levels overtime.**

Patient was sampled overtime after ICU admission and plasma cytokine concentrations were measured by Ella technology and are expressed as pg/mL. References values are provided by routine clinical immunology laboratories at our institution. Interleukin-7 treatment was initiated at day 24 after ICU admission (D24). Cytokine measurements were performed once before IL-7 treatment initiation (< D24) and twice a week during 3 weeks after IL-7 treatment initiation. For each sampling time, if several measurements were performed, the highest value was systematically considered.

|  | **< D24** | **D24**  IL-7 onset | **D27-D29** | **D30-D32** | **D33-D35** | **D36-D39** | **D40-D43** | **D44-D48** | **Reference values** |
| --- | --- | --- | --- | --- | --- | --- | --- | --- | --- |
| **IL-6** | 75,4 |  | 171 | 71,1 | 65,1 | 101 | 70,1 | 30,9 | < 7 |
| **TNF-α** | 24 |  | 38,8 | 37 | 42,2 | 47,6 | 42 | 28,5 | < 10 |
| **IL-1β** | <4 |  | <4 | <4 | <4 | NA | NA | NA | 0-10 |
| **IL-10** | 11,4 |  | 20,9 | 10 | 15,7 | 18,7 | 15,6 | 13,3 | < 8 |
